# Nanofiber-Based Delivery of Bioactive Lipids Promotes Pro-regenerative Inflammation and Enhances Muscle Fiber Growth After Volumetric Muscle Loss

**DOI:** 10.3389/fbioe.2021.650289

**Published:** 2021-03-19

**Authors:** Cheryl L. San Emeterio, Lauren A. Hymel, Thomas C. Turner, Molly E. Ogle, Emily G. Pendleton, William Y. York, Claire E. Olingy, Alan Y. Liu, Hong Seo Lim, Todd A. Sulchek, Gordon L. Warren, Luke J. Mortensen, Peng Qiu, Young C. Jang, Nick J. Willett, Edward A. Botchwey

**Affiliations:** ^1^Department of Biomedical Engineering, Georgia Institute of Technology, Atlanta, GA, United States; ^2^Regenerative Bioscience Center, Rhodes Center for ADS, University of Georgia, Athens, GA, United States; ^3^School of Mechanical Engineering, Georgia Institute of Technology, Atlanta, GA, United States; ^4^Petit Institute for Bioengineering and Bioscience, Georgia Institute of Technology, Atlanta, GA, United States; ^5^School of Biological Sciences, Georgia Institute of Technology, Atlanta, GA, United States; ^6^Department of Physical Therapy, Georgia State University, Atlanta, GA, United States; ^7^School of Chemical, Materials, and Biomedical Engineering, University of Georgia, Athens, GA, United States; ^8^Department of Orthopedics, Emory University, Atlanta, GA, United States; ^9^Atlanta Veterans Affairs Medical Center, Decatur, GA, United States

**Keywords:** immunomodulation, inflammation, tissue engineering, regeneration, sphingolipid

## Abstract

Volumetric muscle loss (VML) injuries after extremity trauma results in an important clinical challenge often associated with impaired healing, significant fibrosis, and long-term pain and functional deficits. While acute muscle injuries typically display a remarkable capacity for regeneration, critically sized VML defects present a dysregulated immune microenvironment which overwhelms innate repair mechanisms leading to chronic inflammation and pro-fibrotic signaling. In this series of studies, we developed an immunomodulatory biomaterial therapy to locally modulate the sphingosine-1-phosphate (S1P) signaling axis and resolve the persistent pro-inflammatory injury niche plaguing a critically sized VML defect. Multiparameter pseudo-temporal 2D projections of single cell cytometry data revealed subtle distinctions in the altered dynamics of specific immune subpopulations infiltrating the defect that were critical to muscle regeneration. We show that S1P receptor modulation via nanofiber delivery of Fingolimod (FTY720) was characterized by increased numbers of pro-regenerative immune subsets and coincided with an enriched pool of muscle stem cells (MuSCs) within the injured tissue. This FTY720-induced priming of the local injury milieu resulted in increased myofiber diameter and alignment across the defect space followed by enhanced revascularization and reinnervation of the injured muscle. These findings indicate that localized modulation of S1P receptor signaling via nanofiber scaffolds, which resemble the native extracellular matrix ablated upon injury, provides great potential as an immunotherapy for bolstering endogenous mechanisms of regeneration following VML injury.

## Introduction

Though skeletal muscle possesses robust potential for healing after injury, large volumetric wounds that occur during combat, accidents or surgical resection often do not heal completely, resulting in fibrotic scarring and limited range of motion (Garg et al., 2015). Current standard of care involves the autologous transfer of tissue but this treatment produces limited functional restoration and also results in complications at both the donor and injury site (Beth and Pollot, 2016). Moreover, extensive soft tissue injury and concomitant damage to collateral blood vessels results in inadequate vascularization to the regenerating or grafted tissue. Recently, this unsuccessful muscle regeneration outcome following a traumatic injury has been linked to a dysfunctional immune response. Specifically, the immune response is overwhelmed by pro-inflammatory myeloid cells that exacerbate the pro-inflammatory phase of inflammation to a point where regeneration is not able to take place. Instead, the prolonged pro-inflammatory phase induces fibrosis and long-term loss of function (Aguilar et al., 2018; Larouche et al., 2018).

Monocytes and macrophages have become increasingly recognized as key regulators of microenvironmental cues within injured skeletal muscle (Arnold et al., 2007; Saclier et al., 2013). Tightly coordinated signals from monocyte/macrophages subpopulations govern the endogenous muscle stem cell (MuSC) niche and dictate the dynamic switch between either regenerative or fibrotic healing outcomes. Monocytes and macrophages are highly plastic immune cells that display a range of phenotypes *in vivo*. The classical, inflammatory monocyte is Ly6C^hi^ whereas non-classical, anti-inflammatory monocytes are Ly6C^lo^ (Geissmann et al., 2003). Ly6C^hi^ monocytes are rapidly recruited to the injury, where they can convert *in situ* into Ly6C^lo^ monocytes and become the primary contributors toward polarizing the macrophage population to a pro-regenerative (“M2”) phenotype instead of an inflammatory (“M1”) phenotype (Arnold et al., 2007). The presence of M2 macrophages marks the beginning of the regenerative phase of muscle healing; during this stage, M2 macrophages secrete IGF-1 to promote MuSC proliferation and subsequently secrete key growth factors, including growth differentiation factor 3 (GDF3), to promote myogenic differentiation (Tonkin et al., 2015; Juban and Chazaud, 2017). We have previously demonstrated that non-classical anti-inflammatory Ly6C^lo^ monocytes express relatively high levels of sphingosine-1-phosphate receptor 3 (S1PR3), which can be leveraged to modulate their response *in vivo* to promote the onset of the regenerative phase of muscle healing (Awojoodu et al., 2013).

Sphingosine-1-phosphate (S1P), a product of membrane sphingolipid metabolism, is a pleiotropic bioactive signaling lipid that activates signaling through five known G protein-coupled receptors, S1PR1-5 (Rivera et al., 2008). Extracellular gradients of S1P, achieved through the use of S1P chaperones, S1P neutralizing agents, sphingolipid metabolic enzymes, and biased agonists or antagonists of S1PR isotypes, can control fundamental processes such as immune cell fate and vascular integrity (Cartier and Hla, 2019). Additionally, the S1P signaling axis has been implicated in stimulating proliferation, motility and survival of MuSCs and inducing myogenic differentiation of myoblasts (Donati et al., 2013). Thus, S1P receptors have become an attractive pharmacological target in various autoimmune and inflammatory diseases including those affecting muscle repair and regeneration. This is perhaps most evident with the synthesis of Fingolimod (FTY720) from myriocin and its subsequent passing of all clinical trials and approval for the treatment of relapsing-remitting multiple sclerosis (Kappos et al., 2006). FTY720 is a small molecule agonist of S1PR1 and S1PR3-5 along with being a selective functional antagonist of S1PR1 by inducing irreversible S1PR1 internalization and degradation (Maceyka et al., 2012). We have shown previously that biomaterial-based gradients of S1P are short-lived in the tissue due to S1P degradation by S1P lyase. However, the active, phosphorylated form of FTY720, FTY720-P, is not catabolized by S1P lyase and can undergo multiple rounds of signaling due to its ability to be reversibly phosphorylated; this provides a more stable and sustained gradient in the tissue as observed in our previous work (Ogle et al., 2014). We have shown that local delivery of FTY720 recruits non-classical Ly6C^lo^ monocytes upon local delivery to inflamed skin injuries and promotes microvascular growth in an S1PR3-dependent manner (Olingy et al., 2017). Release of FTY720 from polymer nanofibers to mandibular bone defects increases the frequency of Ly6C^lo^ anti-inflammatory monocytes, promotes re-vascularization, and facilitates boney ingrowth (Das et al., 2013). Thus, the local delivery of an S1PR modulator, like FTY720, has emerged as a small molecule approach that can orchestrate the inflammatory response and subsequent healing of musculoskeletal injuries (Das et al., 2014; San Emeterio et al., 2017; Hymel et al., 2020).

In this series of studies, we investigated the immunomodulatory response and muscle regeneration outcome in a murine volumetric muscle loss (VML) injury after FTY720-loaded nanofiber implantation. The nanofiber implant was manufactured using 50/50 poly(lactic-co-glycolic acid) (PLGA)/Polycaprolactone (PCL) and was characterized using scanning electron microscopy (SEM) and atomic force microscopy (AFM) (Supplementary Figure 1). We used traditional “manually gated” immune populations, where regions of interest (manual gates) were drawn over a series of bivariate plots, for further analysis (Supplementary Figure 1A–I). Due to their ability to resolve subtly different cell populations, we applied both uniform manifold approximation and projection (UMAP) and spanning tree progression of density normalized events (SPADE) for dimension reduction and unbiased cluster identification (Supplementary Figure 1A–II,III) (Becht et al., 2018). SPADE is unique in its ability to reconstruct complex cellular hierarchies of immune cell transitions in order to reveal rare cell states before and after perturbations, such as injury or drug delivery (Qiu et al., 2011). Thus, we took advantage of this utility to classify the nodes of SPADE dendrograms to an immune cell subset and calculated how the cell frequency of these subsets is affected by FTY720 nanofiber treatment (Supplementary Figure 1A–IV,V). In addition to the single-cell analysis we performed whole mount immunohistochemistry to describe the functional muscle healing outcome after FTY720 nanofiber treatment (Supplementary Figure 1B–I). Using two-photon confocal microscopy along with second harmonic generation (SHG) imaging and 3D reconstruction of images using Imaris software, we were able to characterize the muscle fiber alignment, collagen deposition, myeloid cell infiltration and reinnervation of muscle tissue (Supplementary Figure 1B–II,V). We hypothesized that local S1PR signaling modulation via nanofiber-based delivery of FTY720 from nanofiber scaffolds in murine VML defect models would shift the milieu to a more pro-regenerative microenvironment, leading to improved muscle regeneration and thus providing a promising therapeutic strategy to ameliorate the clinical burden VML presents.

## Results

### Characterization of FTY720-Loaded Electrospun Nanofiber Scaffolds

**FIGURE 1:**
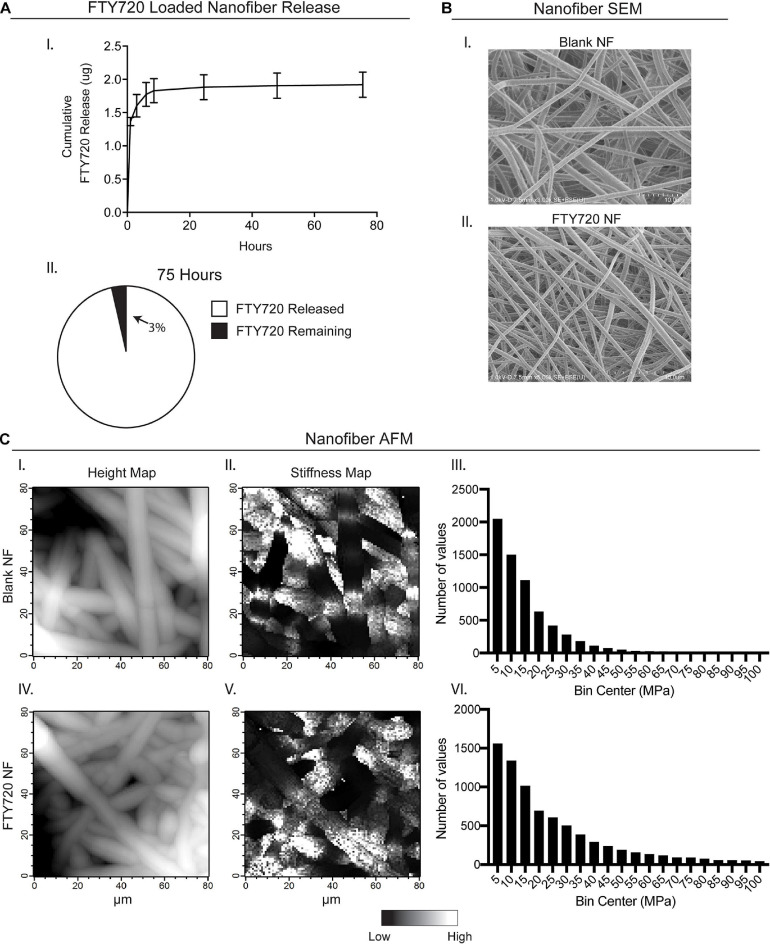
Characterization of electrospun FTY720-loaded nanofiber scaffolds. Release of FTY720 from PLGA-PCL nanofiber (NF) scaffolds and biomaterial characterization via scanning electron microscopy (SEM) and atomic force microscopy (AFM). **(A)** *In vitro* cumulative release profile of FTY720 (I) with percentage of drug released after 75 h (II). **(B)** Scanning electron micrographs of unloaded, blank PLGA-PCL nanofibers (I) and nanofibers loaded with FTY720 (II). **(C)** AFM surface topology (I, IV) and stiffness (II, V) maps of blank and FTY720-loaded nanofibers where white regions represent areas of greater height and stiffness, respectively. Histograms of Young’s moduli (MPa) obtained over multiple contact points on blank nanofibers (III) and FTY720-loaded nanofibers (VI).

In order to provide a biomaterial-mediated release of S1PR modulator, FTY720, to the local injury milieu, we fabricated PLGA/PCL nanofibers via electrospinning. FTY720 was released from the nanofiber scaffolds *in vitro* for 75 h, with approximately 96% of drug release occurring within the first 24 h (Figure 1A–I,II). After 75 h, about 97% of incorporated FTY720 had been released with only 0.065 ± 0.004 μg remaining in the scaffold. SEM and AFM were utilized to assess the morphology and mechanical properties between unloaded, blank nanofibers and those loaded with FTY720 to ensure that any differences in healing outcomes after injury were not attributed to variations in biomaterial characterization (Figures 1B,C). Scanning electron micrographs of blank nanofibers (Figure 1B–I) revealed a similar randomly oriented morphology to those loaded with FTY720 (Figure 1B–II). To evaluate the surface of the nanofibers with high resolution, we performed AFM to confirm that the topography of FTY720-loaded nanofibers was consistent with those that were not loaded with an immunomodulatory drug (Figure 1C–I,IV). Further, Young’s modulus values were obtained over multiple contact points on the nanofibers, showing similar distribution profiles between unloaded and loaded scaffolds (Figure 1C–II,III,V,VI). These techniques establish that both surface and mechanical properties of blank versus FTY720-loaded nanofibers were unaffected by incorporation of this S1P analog, consistent with our previous studies utilizing nanofibers loaded with small molecule S1P receptor modulators (Hymel et al., 2020).

### Localized S1P Receptor Modulation Primes the Injury Milieu for Inflammation Resolution

In order to study how local modulation of S1P receptors impacts timely resolution of inflammation and tissue regeneration, we made a 2 mm full-thickness defect to the murine spinotrapezius muscle (Supplementary Figures 2A,B). This VML model enables whole-mount visualization of cell populations and tissue microstructures involved in muscle repair. Following injury, mice were either left untreated, or underwent implantation of 3 mm blank or FTY720-loaded nanofiber scaffolds over the defect area (Supplementary Figure 2C). Because systemically administered FTY720 is known to induce lymphopenia by suppression of lymphocyte egress from lymph nodes (Blaho and Hla, 2014), we sought to determine whether localized administration of the drug resulted in any system-level effects. Analysis of blood lymphocyte populations by flow cytometry 1-day post injury revealed that both CD4^+^ and CD8^+^ T cell subsets were significantly decreased in the circulation of FTY720 nanofiber treated animals compared to no implant controls yet returned to similar levels by day 3 post injury (Supplementary Figures 3A,B). Systemic Ly6C^hi^ inflammatory monocytes were not affected by local FTY720 release, but Ly6C^lo^ anti-inflammatory monocytes showed a relative increase in the blood at later timepoints (Supplementary Figures 3C,D).

**FIGURE 2:**
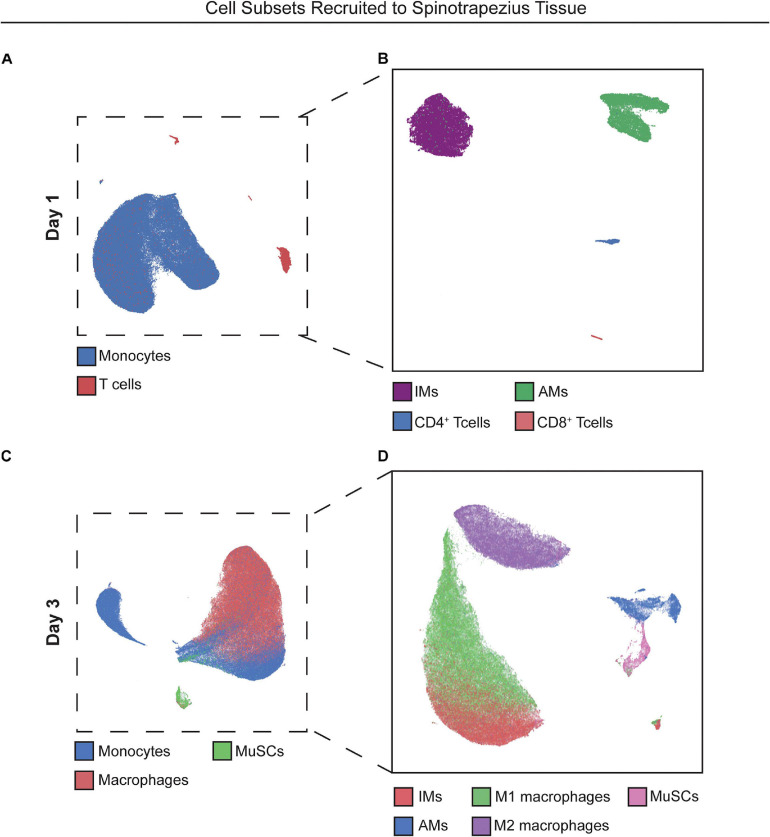
UMAP projections reveal heterogeneous cell subpopulations present in spinotrapezius tissue 1 and 3 days after injury. 2D UMAP projections of single cells taken from explanted spinotrapezius tissues 1 and 3 days post injury. **(A)** UMAP of monocytes and T cells within the muscle at day 1 after injury where these larger cell types are broken down into their respective subpopulations (Ly6C^hi^ and Ly6C^lo^ monocytes, CD4^+^ and CD8^+^ T cells) in a separate UMAP **(B)**. **(C)** Monocyte, macrophage, and MuSCs comprise the cell composition of muscle tissue 3 days post injury with monocyte and macrophage cells types further examined for heterogeneous subpopulations in panel **(D)**.

We hypothesized that local targeting of S1PR1 within the injury niche would prevent over accumulation of inflammatory immune cells by regulating timely tissue egress while simultaneously participating in the recruitment and polarization of pro-reparative cellular players. We performed flow cytometry of cells extracted from the muscle tissue 1 and 3 days after critical injury. At 1-day post injury, recruited monocytes overwhelm the immune cell population within the injured muscle. The heterogeneity underlying the monocyte population, based primarily on Ly6C expression, is demonstrated by their distant island locations within 2D UMAP projections. Distinct T cell islands distinguishing CD4 and CD8 antigen expression were also illustrated by UMAP analysis, although T cells were largely not present in the injured tissue at day 1, likely due to suppressed lymphocyte egress resulting from functional antagonism of S1PR1 (Figures 2A,B).

**FIGURE 3:**
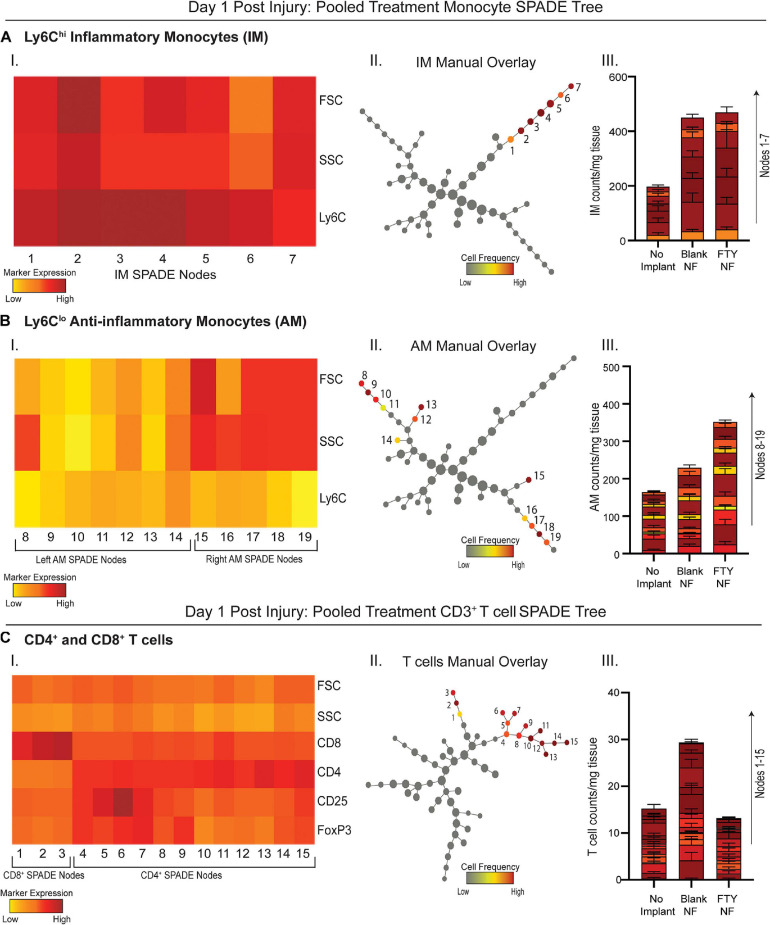
Pseudotime analysis of immune cell infiltration into muscle defect area 1-day post VML injury. SPADE dendrograms consist of live, single cellular events gated as monocytes **(A,B)** and T cells **(C)** extracted from spinotrapezius muscle 1 day after VML injury from all treatment groups. Specific pre-gated cell subpopulations are overlaid onto their respective SPADE dendrograms where overlaid cells are color annotated by normalized cell frequency within the SPADE nodes. SPADE nodes of high frequency of a particular cellular subset (color annotated) are given a node number assignment to present a grouping of nodes distinguishing a certain cell subpopulation within the greater dendrogram trajectory (**A–C** II). The protein signature of the annotated SPADE nodes representing a specific subpopulation of cells is provided as a heatmap (**A–C** I). The number of cells in a given subpopulation per mg of tissue across all treatment groups quantified per annotated SPADE node and represented with a stacked bar graph where each segment of the bar graph corresponds to a given node number assignment (**A–C** III). Data presented as mean ± S.E.M per annotated node where all annotated nodes for a specified subset are stacked vertically in a bar graph for each treatment group. Statistical analysis performed includes one-way ANOVA with Tukey’s multiple comparison. *n* = 6 samples for FTY720 and blank nanofiber (NF) groups and four samples for no implant (untreated) group.

Because traditional strategies for analyzing multiparameter flow cytometry data are prone to user error and limited in its capacity to visualize the correlations among several markers from a series of bi-plots, the complexity of immune cell states may be severely undermined. We employed an unbiased clustering algorithm (SPADE) which preserves the inherent structure of the data and generates nodes to represent clusters of cells with similar marker expression. These nodes are positioned along an inferred trajectory, or “pseudotime” in order to visualize dynamics changes in (pseudo)temporal biological processes, such as cellular transitions, when longitudinal time series data is not available. To assess how local delivery of FTY720 biases immune cell phenotype at early timepoints, we generated SPADE dendrograms made of cells extracted from the muscle tissue 1 day after injury (Figure 3). SPADE dendrograms are comprised of live, single cells gated as CD11b^+^CD64^+^MerTK^–^ monocytes (Figures 3A,B) or CD3^+^ T cells (Figure 3C) from untreated (no implant) animals as well as those treated with blank or FTY720-loaded nanofiber scaffolds. The SPADE dendrograms were further annotated by overlaying manually gated monocyte or T cell subsets, including Ly6C^hi^ inflammatory and Ly6C^lo^ anti-inflammatory monocytes onto the greater monocyte dendrogram (Figures 3A,B, respectively) in addition to CD4^+^ and CD8^+^ T cell subsets overlaid onto the T cell dendrogram (Figure 3C). Nodes in the SPADE tree are colored in a gradient of cell frequency to illustrate where each manually overlaid cellular subset resides. For the pooled treatment monocyte SPADE dendrogram, Ly6C^hi^ inflammatory monocytes are annotated as nodes 1–7 (Figure 3A–II) while Ly6C^lo^ anti-inflammatory monocytes are annotated as nodes 8–19 (Figure 3B–II), each subset clustering in different SPADE nodes and trajectories to demonstrate their unique protein signatures. These protein signatures are further represented with SPADE node heatmaps that graphically represent heterogenous marker expressions characteristic of each monocyte subset (Figures 3A–I, B–I). For each identified monocyte subpopulation, we generated stacked bar graphs to visualize cell frequency differences within annotated nodes amongst treatment groups (Figures 3A–III, B–III). Each segment of the bar graphs corresponds to an annotated SPADE node (colored and stacked numerically by node) for direct comparison of cell frequency in nodes across treatments.

No changes in cell frequency of Ly6C^hi^ inflammatory monocytes were evident across treatment groups at day 1, whereas local FTY720 delivery resulted in an early relative increase of Ly6C^lo^ anti-inflammatory monocytes compared to blank nanofiber and no implant control groups (Figures 3A–III, B–III). We have previously shown that these Ly6C^lo^ anti-inflammatory monocytes are biased progenitors of regenerative M2-like macrophages and are critical to regenerative processes in other tissues (Olingy et al., 2017; San Emeterio et al., 2017). Manually gated CD8^+^ T cells were overlaid onto the pooled treatment CD3^+^ SPADE dendrogram, located in nodes 1–3, while CD4^+^ helper T cells were positioned in nodes 4–13 (Figure 3C–II). Distinct dendrogram locations between these T cell subsets result from their protein expression differences that is visualized in an accompanying annotated SPADE node heatmap (Figure 3C–I). While collective CD4^+^ and CD8^+^ T cell numbers in injured tissue was not significantly different across treatments, we note that FTY720 treated animals had reduced T cell infiltration compared to those treated with blank nanofibers (Figure 3C–III). This was presumably a result of the acute lymphopenia induced at day 1 (Supplementary Figure 3). These results indicate that while immune cell recruitment into the tissue microenvironment was not yet significantly affected by therapeutic S1P receptor modulation at day 1 post injury, we see rising pro-regenerative monocyte subsets and decreased lymphocyte invasion which may play a vital role in priming the injury niche toward timely resolution of inflammation.

### Nanofiber-Based Delivery of a Pharmacological S1P Analog Enriches Pro-regenerative Immune Subsets and MuSC Population at 3 Days Post Injury

With local S1P receptor modulation beginning to shift the injury microenvironment from pro-inflammatory to pro-regenerative at day 1, we aimed to evaluate the therapeutic effect on key cellular players underlying ongoing muscle regeneration at 3 days post injury. Single cells were extracted from muscle tissue at 3 days post injury and analyzed by flow cytometry. Referring back to Figure 2, UMAP projections were again generated with cells extracted from injured muscle at day 3 (Figures 2C,D) and overlaid with select manually gated subsets to visualize the population of monocytes (blue) and macrophages (red) along with a group of resident MuSCs (green) present in the injured muscle (Figure 2C). Figure 2D shows the phenotypic variations between these immune populations, as the distance between M1 and M2-like macrophage clusters and between Ly6C^hi^ inflammatory and Ly6C^lo^ anti-inflammatory monocyte groupings indicate the innate protein expression differences distinguishing subsets existing within bulk cell types.

**FIGURE 4:**
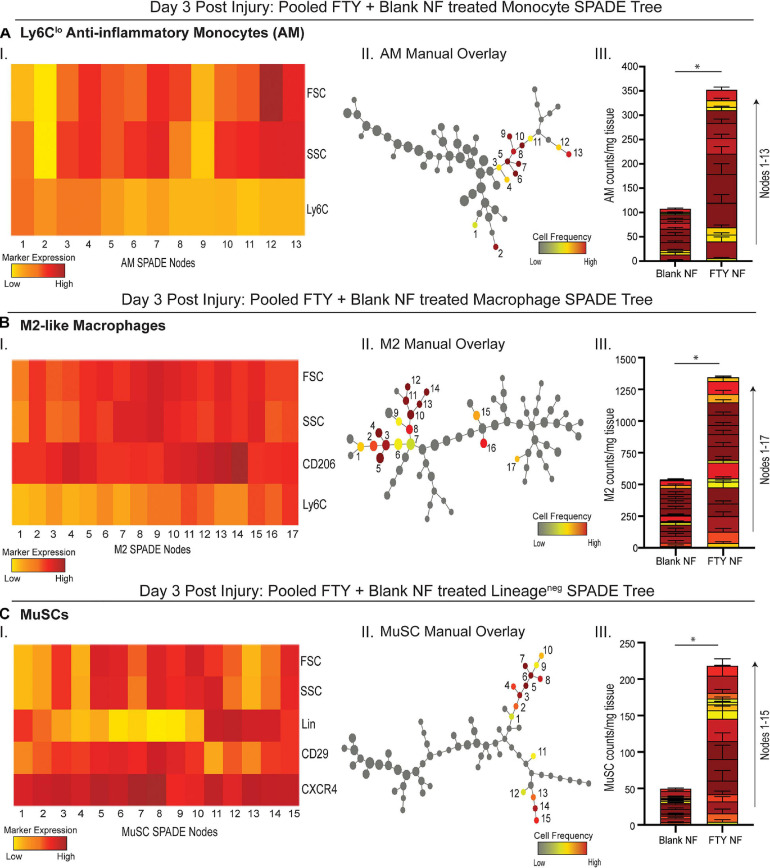
Pseudotime visualization of cellular composition at the site of injury reveals an FTY720-induced increase in pro-regenerative subsets 3 days post VML injury. SPADE dendrograms consist of live, single cellular events gated as monocytes **(A)**, macrophages **(B)**, and Lin^−^ cells **(C)** extracted from spinotrapezius muscle 3 days after VML injury from animals treated with blank or FTY720-loaded nanofibers (NF). Specific pre-gated cell subpopulations (Ly6C^lo^ anti-inflammatory monocytes, M2-like macrophages, and MuSCs) are overlaid onto their respective SPADE dendrograms where overlaid cells are color annotated by normalized cell frequency within the SPADE nodes. SPADE nodes of high frequency of a particular cellular subset (color annotated) are given a node number assignment to present a grouping of nodes distinguishing a certain cell subpopulation within the greater dendrogram trajectory (**A–C** II). The protein signature of the annotated SPADE nodes representing a specific subpopulation of cells is provided as a heatmap (**A–C** I). The number of cells in a given subpopulation per mg of tissue across all treatment groups quantified per annotated SPADE node and represented with a stacked bar graph where each segment of the bar graph corresponds to a given node number assignment (**A–C** III). Data presented as mean ± S.E.M per annotated node where all annotated nodes for a specified subset are stacked vertically in a bar graph for each treatment group. Statistical analysis performed includes unpaired *t*-tests. *n* = 8 samples per treatment group. ^∗^*p* < 0.05.

To evaluate more discrete heterogeneities within subpopulations of cells infiltrating the site of injury in response to S1P receptor agonism at a later stage in the initial inflammatory cascade, we generated SPADE dendrograms consisting of single, live cells from muscle tissue 3 days post VML injury (Figure 4). These SPADE dendrograms provide visual 2D representations of the heterogeneity within pre-gated monocytes (Figure 4A), macrophages (Figure 4B), and Lineage (Lin)^–^ cells (Figure 4C) from animals treated with either blank or FTY720-loaded nanofibers. Select gated populations of interest (Ly6C^lo^ anti-inflammatory monocytes, M2-like macrophages, and MuSCs) were overlaid onto their corresponding SPADE dendrograms (Figures 4A–II,B–II,C–II, respectively). Any node which occupies cells of the overlaid subset is annotated via a node assignment number and color coded by cell frequency within the given SPADE dendrogram. Ly6C^lo^ anti-inflammatory monocytes occupy nodes labeled 1–13 within the monocyte SPADE dendrogram (Figure 4A–II), and their low Ly6C signature is confirmed by its accompanying SPADE node heatmap (Figure 4A–I). Stacked SPADE node bar graphs show that localized nanofiber-mediated release of FTY720 drastically increased the number of Ly6C^lo^ anti-inflammatory monocytes infiltrating the tissue compared to blank nanofiber controls (Figure 4A–III) without any significant difference in the number of Ly6C^hi^ inflammatory monocytes between treatments (Supplementary Figure 4). We further observed significantly more pro-regenerative M2-like macrophages, occupying nodes 1–17 in the macrophage dendrogram (Figure 4B–II), from FTY720 treated animals compared to those treated with blank nanofiber scaffolds (Figure 4B–III). Because macrophages are known to play a role in MuSC proliferation and differentiation, we identified CXCR4^+^ CD29^+^ MuSCs (Figure 4C–I) and overlaid these resident muscle cells onto the Lin^–^ SPADE dendrogram (Figure 4C–II). We found that modulating S1P receptors locally within the muscle tissue significantly enriched resident MuSCs (Figure 4C–III) which is a vital component to successful regeneration given that VML injury results in a catastrophic loss of the MuSC niche (Wosczyna and Rando, 2018). Our results suggest that FTY720 demonstrates its effectiveness as an immunomodulatory therapy after critical injury not only through its recruitment of pro-resolving immune subsets, but additionally by increasing the MuSC numbers four-fold within the injury environment.

### S1P Receptor Immunotherapy Leads to Improved Metrics of Skeletal Muscle Regeneration

We assessed regeneration of muscle fibers 14 days post VML injury with confocal imaging to determine if the observed FTY720-induced immune cell recruitment dynamics underlie overall muscle repair and regeneration. Whole mount confocal images of injured spinotrapezius muscles were stained with desmin (green) to identify muscle fibers and CD68 (blue) to identify macrophages within and around the site of injury; representative images across all treatments groups were rendered in Imaris for 3D visualization of muscle fiber healing (Supplementary Figure 5). While untreated animals and those treated with blank nanofibers presented with large defect areas remaining 14 days post VML injury, muscle treated locally with FTY720 demonstrated accelerated wound healing with regenerating myofibers bridging across the defect area (Supplementary Figures 5A–C). A side view of the 3D muscle renderings showed the qualitative differences notable in CD68^+^ macrophage infiltration into the defect area with untreated and blank nanofiber groups overwhelmed with persistent immune cell presence characterizing chronic, non-resolving inflammation (Supplementary Figures 5D,E). To the contrary, FTY720 delivery to the injured muscle resulted in qualitatively less macrophage crowding in and around the defect (Supplementary Figure 5F), indicating that S1PR modulation plays a pivotal role in efficient immune cell egress from the tissue in a timely manner.

**FIGURE 5:**
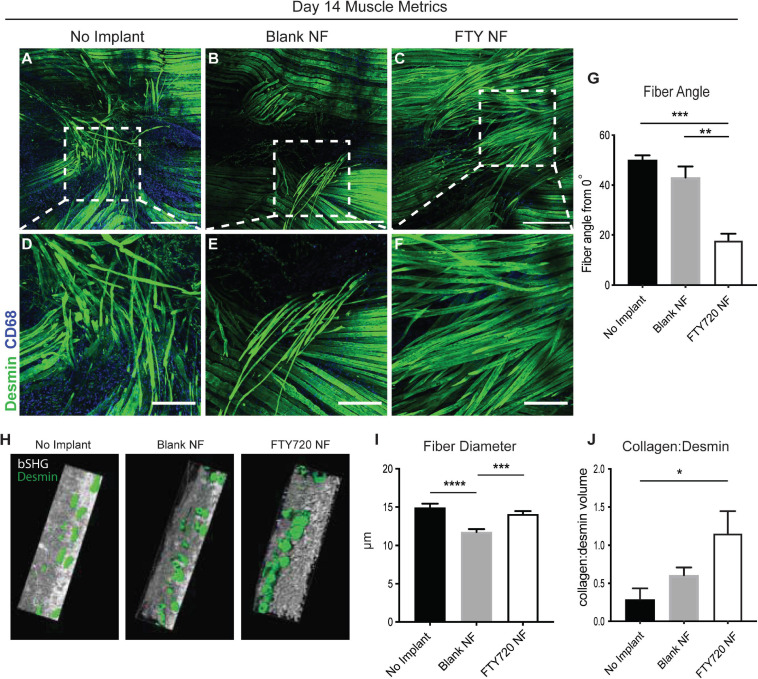
Localized release of S1P analog from nanofiber scaffold enhances metrics of skeletal muscle healing and regeneration. Whole mount IHC of explanted spinotrapezius muscle 14 days post injury. Representative confocal images centered around the defect area from each treatment group stained with desmin (green) and CD68 (blue) **(A–C)** with a zoomed in ROI from each treatment group, denoted with dotted white boxes, presented in the second row **(D–F)**. **(G)** Fiber angle of regenerating muscle fibers measured from 0 to 90° degrees with respect to the pre-injury fiber axis. **(H)** Imaris generated cross-sectional view of regenerating muscle fibers for assessment of fiber morphology. **(I)** Fiber diameter measurements from animals across all treatment groups. **(J)** Volume ratio of collagen:desmin quantified for animals of each treatment group. Scale bars of 500 μm **(A–C)** and 200 μm **(D–F)**. Ordinary one-way ANOVA with Tukey’s multiple comparisons conducted for statistical analysis and data is presented as mean ± S.E.M. *n* = 3–5 animals per treatment group. *p < 0.05, **p < 0.01, ***p < 0.001, ****p < 0.0001. bSHG = backward second harmonic generation, NF = nanofiber.

We then examined the relationship between regenerating muscle fibers and their alignment with healthy fibers during injury repair (Figure 5). We measured the angle between centrally nucleated regenerating muscle fibers and the axis of original, uninjured muscle fibers (denoted in Supplementary Figure 6); a higher angle measurement (ranging from 0 to 90°) indicates poor alignment of the new muscle fibers. Untreated and unloaded nanofiber groups presented with disorganized fibers in random orientations (Figures 5A,B,D,E). Defects treated with FTY720 showed orderly elongation of myofibers across the injury site (Figures 5C,F). FTY720-treated defects exhibited significantly lower angle measurements (17.82° ± 5.4°) compared to both untreated and blank nanofiber treated defects (50.07° ± 3.2° and 43.1° ± 8.79°, respectively), indicating improved alignment of newly developed myofibers (Figure 5G). Surface rendered cross sections of the regenerating muscle fibers surrounding the defect space showed that in addition to enhanced alignment, FTY720 treated fibers had improved circular morphology along with significantly increased fiber diameter compared to untreated and blank nanofiber groups (Figures 5H,I). These improved regenerative metrics observed in muscle treated with immunomodulatory FTY720 could be in part attributed to increased collagen deposition around new myofibers in the FTY720 treated animals (Figure 5J).

**FIGURE 6:**
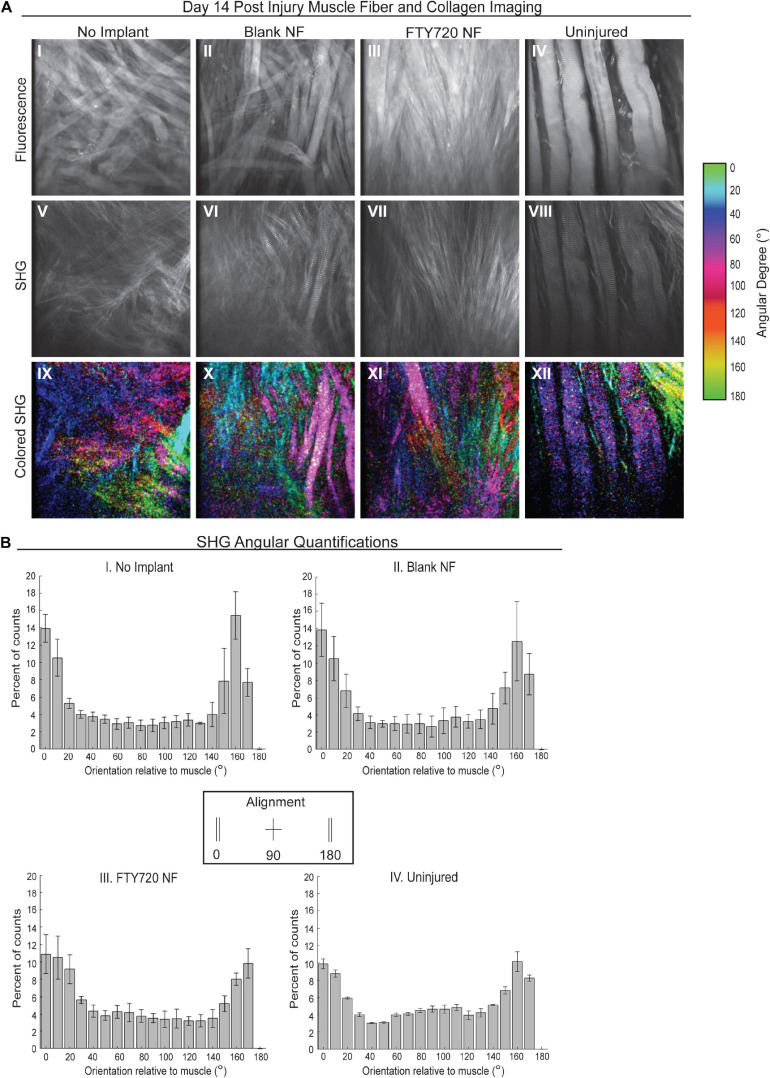
Second harmonic generation (SHG) reveals collagen alignment in regenerating muscle fibers when treated with FTY720-loaded nanofiber scaffolds. **(A)** Second harmonic imaging of muscle fibers from untreated muscle, blank nanofiber (NF) treated muscle, FTY720-loaded nanofiber treated muscle, and uninjured muscle. Representative fluorescence images from each treatment group shown in the first row of images (I–IV), SHG imaging of the same animals per treatment shown in the second row (V–VIII), and the last row represents a color-coded maximum intensity projection in which the color key is annotated based on angular orientation (IX–XII). **(B)** Angular quantifications of collagen in relation to the muscle fiber axes measured via SHG. *n* = 4 per treatment group and data represented as mean ± S.D. SHG = second harmonic generation.

To further probe how collagen and muscle fibers align as the defect heals, we performed SHG imaging 2 weeks post injury to analyze the angular differences between the dominant axes of collagen and muscle fiber orientation (Figure 6). We found that animals treated with FTY720-loaded nanofibers displayed collagen fibrils highly aligned with one another and with the regenerating muscle fibers within the defect area which was not observed in the untreated or blank nanofiber control groups (Figure 6A). In representative images, the maximum intensity angles of SHG signal are color coded for polarimetric distinction between collagen and muscle fibers where purple represents the actin/myosin banding and green represents areas of collagen alignment with the muscle fibers. We graphed the orientation angle of each pixel within the image with orientations of 0 and 180 aligned with the main direction of the muscle fibers. Distribution of the pixel orientations in each field of view had similar shapes in the uninjured and FTY720-treated animals, confirming collagen and muscle fiber alignment during the regenerative process (Figure 6B). Thus, increased collagen deposition induced by FTY720 may be vital to providing a remodeled extracellular matrix and an architectural conduit along which fibers can elongate in an aligned manner vital to mature skeletal muscle structure and function.

### FTY720 Delivery Stimulates Re-vascularization and Regeneration of Damaged Neuromuscular Junctions

**FIGURE 7:**
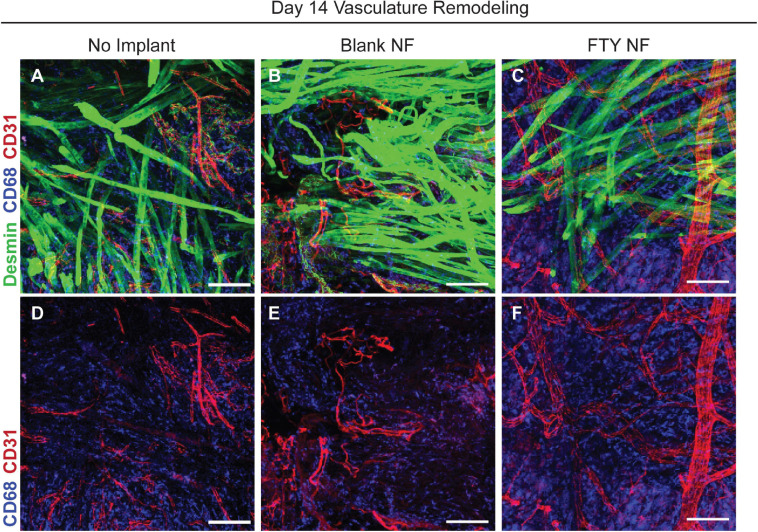
Delivery of FTY720 stimulates re-vascularization within injured skeletal muscle. Whole mount IHC imaging of explanted spinotrapezius muscle 14 days post injury. Representative images within the defect space shown for each experimental condition stained for desmin (green), CD68 (blue), and CD31 (red) to identify muscle fibers, macrophages, and vasculature, respectively **(A–C)**. Second row of images **(D–F)** only depict CD68 and CD31 staining to clearly visualize the vascular network underlying regenerating muscle fibers after VML injury. Scale bars of 100 μm in each image. *n* = 2–3 animals per treatment group.

Restoration of functional properties of skeletal muscle relies on the revascularization and reinnervation of regenerated myofibers, two major challenges to a full recovery following traumatic injury. Representative images of muscle defects across all experimental conditions were stained for desmin (green), CD68 (blue), and CD31 (red) in order to characterize the vascular network present at 14 days post injury (Figure 7). Whereas untreated and blank nanofiber treated animals displayed an immature vascular network of disorganized small-diameter vessels (Figures 7A,B,D,E), FTY720 nanofiber treated animals were distinguished by distinctly formed, branching vasculature amongst regenerating fibers (Figures 7C,F). This result demonstrates the temporal coupling of myogenesis with vascular remodeling processes crucial for successful skeletal muscle regeneration (Borselli et al., 2010; Latroche et al., 2017).

**FIGURE 8:**
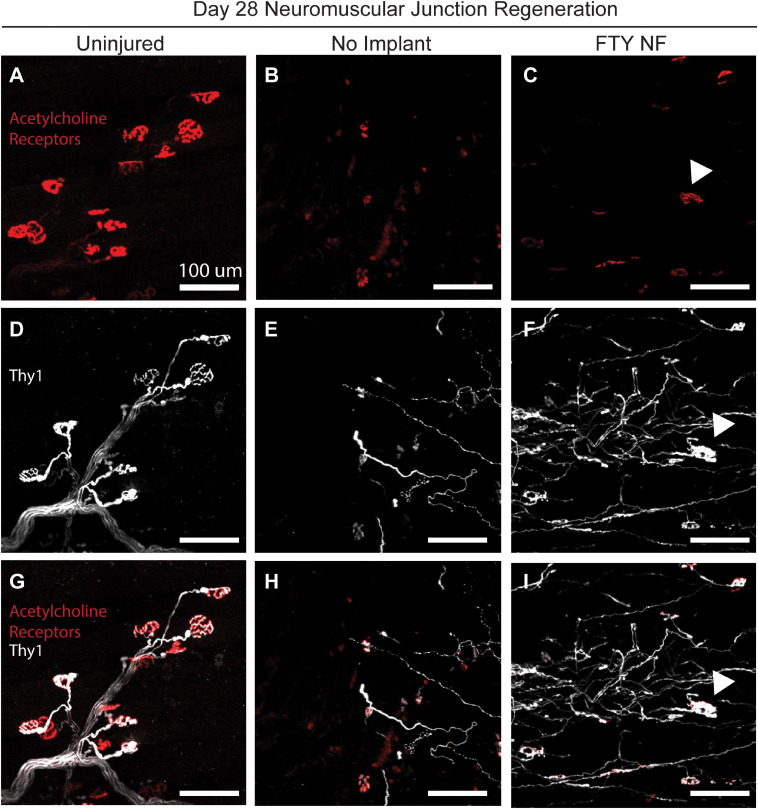
Localized FTY720 delivery induces regeneration of damaged neuromuscular junctions following traumatic injury to the muscle. High powered immunofluorescence images of spinotrapezius muscle explanted 28 days post injury from uninjured muscle **(A,D,G)**, untreated muscle **(B,E,H)**, and muscle treated with FTY720-loaded nanofibers **(C,F,I)**. Thy1-YFP mice enabled labeling of motor neurons with YFP (white) and staining with rhodamine alpha-bungarotoxin (red) permits identification of post-synaptic acetylcholine receptors. Regenerated neuromuscular junctions identified by detecting overlapping regions of Thy1^+^ pre-synaptic and acetylcholine receptor^+^ post-synaptic junctions as indicated with white arrow. Scale bars of 100 μm. *n* = 2–3 animals per experimental group.

To assess the reinnervation of myofibers around the defect area, FTY720 nanofibers were implanted into VML injured Thy1-YFP mice. In this mouse model, Thy1^+^ motor neurons were labeled with YFP and detectable via immunofluorescence (Figure 8). At the 28 days post injury timepoint, regenerated neuromuscular junctions were detected by identifying regions of overlap of Thy1^+^ pre-synaptic junctions (white) and acetylcholine receptor^+^ post-synaptic junctions (red). While acetylcholine receptor clusters were present in untreated muscle, their morphology appeared fragmented and there was little association with motor axons indicative of frank denervation underlying severe VML trauma (Figures 8B,E,H). However, when treated with immunomodulatory FTY720, acetylcholine receptors appeared to begin recovering the pretzel-like morphology (denoted with white arrow) typical of mature, healthy neuromuscular junctions and displayed greater axonal density compared to untreated muscle (Figures 8C,F). The topological maturation of FTY720-treated acetylcholine receptors combined with an overlapping Thy1^+^ axonal network (Figure 8I) suggests that FTY720 promotes regeneration of damaged neuromuscular junctions as soon as 28 days following severe trauma. The improved vascular network development and neuromuscular junction regeneration seen in animals treated locally with FTY720 highlights the promise of S1P receptor modulation toward boosting endogenous repair mechanisms imperative to functional recovery after severe musculoskeletal injury.

## Discussion

Volumetric muscle defect injuries comprise 50–70% of combat injuries and are the cause of 80% of limb amputations in soldiers (Grogan et al., 2011). The therapeutic options for critically sized muscle defects are few and exhibit poor clinical prognosis due to donor site morbidity, and inadequate integration of donor tissue into the local vascular and motor neural network, resulting in permanent functional deficits and disabilities. While extracellular matrix derived scaffolds have been utilized as void filling substitutes for autografts and promote M2-like macrophage polarization in the injury niche, the reported efficacy is highly variable (Qazi et al., 2015; Dziki et al., 2016). Although there have been several regenerative efforts made toward treating VML, the dysregulated immune response following critical VML injury is poorly understood. Subcritical, healing muscle defects exhibit an initial inflammatory phase, with pro-inflammatory immune cells such as Ly6C^hi^ monocytes and M1-like macrophages peaking around days 2–3 post injury. Following this stage, M2-like macrophages and their biased progenitors, Ly6C^lo^ monocytes, will predominate around 4–7 days post injury, marking the resolution of inflammation and bringing forth the restorative phase (∼8–14 days post injury) in which angiogenesis and matrix deposition coupled with MuSC differentiation contribute to desirable muscle healing (Chazaud, 2020). In contrast, critically sized VML injuries, as studied here, present with a dysregulated inflammatory cascade (Supplementary Figure 7). This often leads to chronic inflammation that does not successfully resolve which may result in pathological fibrosis instead of functional recovery. Employing small molecule, regenerative immunotherapies which influence the phenotype and function of endogenous pro-regenerative cell types represents a promising strategy for local immunomodulation of innate and adaptive immune response following critical VML.

In this study, we utilized a murine spinotrapezius VML model to investigate how local delivery of a pharmacological S1PR modulator influences endogenous cellular reprogramming within the injury milieu to foster enhanced myofiber regeneration coupled with revascularization and reinnervation of the muscle tissue. Nanofiber scaffolds fabricated from biocompatible polymers are an easily tunable platform that enables the incorporation of bioactive molecules suited to mediate S1PR signaling pathways critical to leukocyte trafficking and subsequent tissue repair. The nanofibrillar structure of the scaffolds not only resembles the native extracellular matrix that is lost upon traumatic injury, but further, the nanoscale topography and high surface-to-volume ratio improves interactions with cellular components infiltrating the defect (Das et al., 2013). Here, we engineered PLGA/PCL nanofiber scaffolds encapsulating FTY720, an immunomodulatory molecule known to redirect anti-inflammatory Ly6C^lo^ monocytes from peripheral circulation into injured tissues when locally applied (Awojoodu et al., 2013). We have shown previously that nanofiber topography acted synergistically with FTY720 delivery in critically sized bone defects as evidenced by neovascularization within the defect site and recruitment of endogenous progenitor cells to enhance bone regeneration (Das et al., 2013).

While our study aims to target S1PR signaling to regulate the clearance and regenerative polarization of immune cells following VML, conventional methods of analyzing the immune response severely undermine its functional diversity. Cell populations captured at a single moment in time may include several distinct phenotypic transition states, yet this is often masked by averaging properties existing within bulk cell types and thus losing trends of single cells (Trapnell et al., 2014). New analytical and visualization techniques have been developed to allow greater exploration into the diverse populations of immune cells underlying non-healing injuries and their response to therapeutic perturbations. We harness these technological advancements with SPADE, a dimensionality reduction and pseudotime trajectory tool that allows us to assess the immune cell recruitment and differentiation kinetics within an inflammatory, traumatic defect and investigate myeloid and lymphoid responses in this VML injury to the delivery of FTY720. Specifically, we find that while early Ly6C^hi^ inflammatory monocyte infiltration at day 1 post injury was unchanged between treatment groups, there were increases in the number of Ly6C^lo^ anti-inflammatory monocytes within animals treated with FTY720-loaded nanofibers compared to controls (Figures 3A,B). The Ly6C^hi^ inflammatory and Ly6C^lo^ anti-inflammatory monocytes occupy nodes in different trajectories of the SPADE dendrogram, illustrating SPADE’s ability to distinguish varying protein signatures within bulk cell types (i.e., monocytes) and visualize the immune cell diversity central to wound healing. The upward trend of Ly6C^lo^ anti-inflammatory monocytes at day 1 with FTY720 treatment may account for robust M2-like macrophage increases seen at day 3, as this result aligns with other studies showing S1P’s role in modulating macrophage polarization within the local injury milieu (Weigert et al., 2019).

Furthermore, there was a clear reduction in CD4^+^ and CD8^+^ T cell infiltration at day 1 post injury with FTY720 treatment, likely as a result of irreversible surface S1PR1 internalization and degradation (Figure 3C). Although the role of CD4^+^ and CD8^+^ T cells in volumetric muscle defect healing remains unclear, these cell subsets may secrete cytokines that could influence the polarization of myeloid subsets. CD4^+^ T cells produce IL-4 and IL-13 (Anthony et al., 2007) which may lead to pro-regenerative M2-like macrophage polarization (Spiller et al., 2016) while CD8^+^ lymphocytes secrete INF-γ (Schoenborn and Wilson, 2007), promoting an inflammatory M1-like macrophage phenotype (Spiller et al., 2016). While invasion of both T cell populations was reduced in muscle treated with FTY720, the complex interplay between lymphoid and myeloid cells is such that even slight shifts in cytokine balance due to decreases in these lymphoid populations could results in a tissue microenvironment more favorable to promoting regeneration.

To assess whether the FTY720-mediated decreasing trends in T cell infiltration and simultaneous increasing trends in Ly6C^lo^ anti-inflammatory monocytes at an early day 1 timepoint primes myeloid phenotypes at later timepoints, we again employ novel, pseudotime trajectory profiling of cell types comprising the injury niche at day 3 post VML. With significant increases in Ly6C^lo^ anti-inflammatory monocytes and M2-like macrophages compared to blank nanofiber treatment (Figures 4A,B), it is concluded that pharmacological agonism of the S1P/S1PR signaling axis preferentially recruits immune cells which drive inflammation resolution to ultimately set the stage for successful wound healing. While the transition from M1 to M2-like macrophages is not binary but rather a continuum of phenotypes, innovative high dimensional analytical tools such as SPADE are indispensable to identifying discrete macrophage subpopulations vital to fostering myogenesis. Both M1 and M2-like macrophages are known to influence MuSC proliferation and differentiation, so we decided to evaluate the effect of S1P receptor modulation on MuSC numbers at day 3 after injury. Interestingly, upon treatment with FTY720, injured spinotrapezius muscle harbored four times as many MuSCs compared to blank nanofiber treated muscle (Figure 4C). M1-like macrophages secrete cytokines such as TNFα and INF-γ which stimulate MuSC proliferation. M2-like macrophages promote the ongoing MuSC proliferation via initial IGF-1 secretion, but they also secrete GDF3 and TGFβ to stimulate MuSC differentiation and fusion to mature myofibers (Wosczyna and Rando, 2018). Thus, a tightly regulated switch in macrophage phenotype is critical for timely MuSC proliferation and myofiber maturation. We believe that the FTY720-mediated M1 to M2-like macrophage switch had a direct influence on MuSC differentiation via paracrine signaling, thus representing a vital therapeutic opportunity to intervene in the initial inflammatory cascade. Importantly, we observed similar increases in M2-like macrophages and MuSC numbers following local delivery of VPC01091 (local S1PR3 antagonist) to injured muscle in our previous studies (Hymel et al., 2020). Further studies exploring the cytokine profile after muscle injury in response to S1P receptor modulators are needed to examine the complex paracrine signaling between various myeloid cell subsets with muscle resident progenitor populations driving endogenous repair mechanisms.

To explore whether the immunological profile and increased MuSC numbers induced by FTY720 treatment influenced subsequent muscle repair and regeneration, we assessed various healing parameters. Rendering whole-mount confocal images of the muscle fibers surrounding the defect area into 3D surfaces, we qualitatively observe enhanced myofiber bridging across the defect space in FTY720 treated muscle compared to control groups which still present with large void areas 14 days post injury (Supplementary Figure 5). Intriguingly, there was an obvious decreased accumulation of CD68^+^ macrophages in the FTY720 treated muscle. This finding is supported by our previous work in which the local delivery of S1P receptor modulator VPC01091 to injured muscle also resulted in decreased macrophage infiltration and more regenerating muscle fibers closing the defect area compared to controls (Hymel et al., 2020). We believe that the role of S1P signaling in regulating immune cell egress from non-lymphoid tissues rids the microenvironment of chronic inflammation, thus accelerating repair and regeneration as seen here with FTY720 treatment as well as with VPC0191 treatment (Baeyens and Schwab, 2020; Hymel et al., 2020). We observe that FTY720 nanofiber treated animals displayed increased fiber diameter compared to control animals, and increased collagen:desmin ratio (Figures 5H–J). Though we have previously attributed high collagen:desmin ratio to an increased fibrotic response, those observations were made in 1 mm volumetric defects which heal around 7 days (San Emeterio et al., 2017). In this 2 mm defect model, tissue must regenerate over three-fold more area than the 1 mm defect model, thus increased matrix deposition may be beneficial in this context to guide and support the growth of muscle fibers.

Because skeletal muscle fibers produce maximum force along a single axis, it is crucial for injured fibers to regenerate in alignment with pre-existing, healthy fibers. We observed a lower angle between regenerating fibers and the original fiber axis in animals treated with FTY720-loaded nanofiber scaffolds, indicating improved alignment of the muscle fibers themselves (Figure 5G) and their alignment with the surrounding extracellular matrix (Figure 6). We can conclude that the higher alignment is primarily due to the effects of FTY720, and not the presence of nanoarchitecture within the scaffold, due to the significantly higher fiber angle found in the blank nanofiber group. The mechanism by which FTY720 mediates fiber alignment is unknown at this point. However, M2-like macrophages have been shown to promote extracellular matrix synthesis within fibroblasts (Knipper et al., 2015) and fibroadipogenic progenitors (Lemos et al., 2015), and it has been recently shown that fibroblasts within a collagen matrix rapidly generate tensile forces to close surgically induced defects (Sakar et al., 2016). An interesting hypothesis is that the increase in M2-like macrophages mediated by FTY720 enhances fibroblast activity to generate contractile forces over the defect void that encourage muscle cell alignment as demonstrated *in vitro* (Liao et al., 2008). Further, it is likely that M2-like macrophages play an important role in guiding muscle fiber growth as they secrete matrix metalloproteinases which remodel the surrounding matrix, but further studies are necessary to determine whether specialized macrophage populations play a direct role in regulating newly formed muscle fiber morphology and orientation. Future studies probing matrix remodeling at later time points with FTY720 treated defects will be crucial to ensure that increased matrix deposition remodels sufficiently so that persistent fibrosis does not occur.

Lastly, we investigated whether our small molecule S1PR modulating immunotherapy could enhance revascularization and reinnervation after volumetric defect injury-two of the most prominent clinical barriers to regaining full muscle function. Within the localized injury area, FTY720 treated animals present a distinct vascular network among regenerating fibers compared to the smaller, immature vessels displayed by control groups at 14 days post injury (Figure 7). In evaluating the ability of FTY720 to regenerate damaged neuromuscular junctions, we found that treated animals exhibit greater Thy1^+^ axonal density than untreated animals. Most importantly, these pre-synaptic junctions are spatially associated with morphologically maturing post-synaptic acetylcholine receptors, indicating ongoing regeneration of the motor neural network just 28 days after critical injury which is not observed in the untreated control (Figure 8). While FTY720’s impact in resolving chronic inflammation and promoting MuSC differentiation likely paves the way for successful reinnervation to occur, FTY720 may also act directly on neuromuscular junctions, as previous work has linked FTY720 to directly regulating synaptic function (Darios et al., 2017). While the qualitative assessment of FTY720-induced revascularization and reinnervation of injured muscle is promising, the lack of quantitative analysis remains a limitation of this work, and further studies are necessary to characterize these regenerative outcomes more thoroughly. Further, determining whether improved revascularization and reinnervation mediated by S1PR modulation can translate to greater contractile forces generated by regenerating muscle is an exciting future direction for ensuring successful functional outcomes.

Taken together, our work demonstrates the ability of utilizing biomaterial-mediated delivery of FTY720 to locally modulate the overwhelming and dysregulated immune response characterizing critical volumetric muscle injuries. We show that S1P receptor modulation results in increased pro-regenerative immune cell infiltration into the defect area in the early phases of the inflammatory cascade with simultaneous increases in activated MuSCs vital to regeneration of muscle fibers. By harnessing the capabilities of multiparameter pseudotime technologies, we are able to detect subtle cellular phenotype transitions mediated by therapeutic perturbations that are crucial to priming the injury milieu for enhanced regeneration. Therefore, as the identification and targeting of specific cellular players is critical to developing successful immunotherapies, this S1PR modulating strategy provides a platform for targeting distinct cellular responses with small molecule therapeutics that can be applied to a wide range of injuries and diseases.

## Materials and Methods

### Nanofiber Scaffold Fabrication

Electrospun nanofiber scaffolds were fabricated as previously described (Das et al., 2013). PCL (Sigma) and PLGA were combined in a 1:1 weight/weight ratio at 18% polymer concentration for blank fibers and 20% polymer concentration for FTY720 (Cayman Chemical, crystalline solid formulation) loaded fibers. Polymers were dissolved in a 1:3 volume ratio solution of methanol to chloroform and vortexed for 2+ hour prior to spinning. For FTY720 loaded fibers, FTY720 was weighed out and directly added to the polymer solution at a 1:200 drug: polymer weight ratio. Two mL of either polymer solution was loaded into a 3 mL syringe with diameter of 10 mm. Electrospinning was performed at an applied voltage of 19 kV, and flow rate of 1 mL/h for both blank and FTY720 fibers. Working distance was set at 10 cm for blank fibers and 12 cm for FTY720 fibers. After 2 mL of polymer was spun, fibers were wrapped in low-binding plastic folders and stored at −20°C.

### Drug Release From Nanofiber Scaffolds

Circular biopsy punches were used to punch out 3 mm discs from electrospun nanofiber sheets. Discs were placed in 100 μL of stimulated body fluid containing 4% fatty acid-free bovine serum albumin. Releasate was collected at the defined timepoints and replaced with fresh release medium.

### Mass Spectrometry Quantification of FTY720 Release Samples

Fingolimod was extracted from release samples using a long chain base sphingolipid extraction protocol (Ogle et al., 2014). 100 μL of release media was placed in glass culture tubes with screw caps (tubes: Kimble-Chase 73750-13100, caps: Kimble-Chase 73802-13415) and 50 μL of phosphate buffered saline was added to bring total sample volume to 150 μL. 1.5 mL of 2:1 methanol to dichloromethane (by volume) extraction solvent was added to each tube. 50 picomoles of C17 sphingosine was added to each tube to serve as an internal standard. Tubes were capped, sonicated for 1 min, vortexed briefly, and incubated overnight at 48°C. The following day, tubes were allowed to cool to room temperature. Potassium hydroxide (150 μL of 1N) was added to each tube, sonicated for 1 min, vortexed briefly, and tubes were incubated at 37°C for 2 h. Samples were allowed to cool to room temperature, and 8 μL of glacial acetic acid was added to each tube before being sonicated for 1 min, and vortexed briefly. Each sample was checked to ensure a neutral (7.0) pH. Samples were centrifuged at 3000 rpm and supernatant collected in separate open top glass tubes. Extraction solvent (0.5 mL) was added to each original sample tube, and samples were sonicated for 1 min and vortexed briefly before being centrifuged again at 3000 rpm. The second supernatant was collected and added to first supernatant. Extraction solvent was evaporated off overnight by a vacuum concentrator (SpeedVac). The next day, 300 μL of mass spectrometry analysis solvent was added to each tube before being sonicated for 2 min. Samples were transferred to microcentrifuge tubes and centrifuged at 18,000 × *g* for 10 min. Supernatant (200 μL) was transferred to mass spectrometry sample vials. Samples were analyzed on a Micromass Quattro LC mass spectrometer.

### Force Mapping of S1PR Modulating Nanofibers With AFM

All AFM measurements were performed with a MFP-3D Bio-AFM (Asylum Research, Santa Barbara, CA, United States). Nanofiber scaffolds were prepared as described and fixed on glass slides with double-sided tape. For stiffness measurements, 9.6 μm spherical PMMA particles (modulus ∼3 GPa) were attached to tipless silicon cantilevers (NSC35-C, Mikromasch, Sofia, Bulgaria) using a two-part epoxy and dried for at least 24 h. We use the thermal equipartition method (Hutter and Bechhoefer, 1993) to calibrate the cantilever spring constant immediately prior to use by indenting a glass substrate and performing a Lorentzian fit to the thermal spectrum. Topological images and accompanying stiffness characterizations of nanofibers were obtained from regions of 80 × 80 μm^2^ force maps with 96 × 96 force curves per image. All the force-distance measurements were performed with 2 μm/sec approach velocity using a 500 nN force trigger. Force-distance plots were transformed into force-indentation depth plots and then 10–100% indentation depth was used to evaluate Young’s modulus using a Hertzian contact model.

### SEM of Nanofibers

Nanofiber scaffolds were sputter coated with gold for 30 sec and imaged on a Hitachi SU8010 Scanning Electron Microscope.

### Spinotrapezius Volumetric Muscle Loss Surgery and Nanofiber Scaffold Implantation

All animal procedures were conducted according to protocols approved by Georgia Institute of Technology Institutional Animal Care and Use Committee. Male C57BL/6J (The Jackson Laboratory) of age 8–12 weeks old were used for all animal studies with the exception of neuromuscular regeneration assessment for which B6.Cg-Tg (Thy1-YFP)16Jrs/J (Jackson Laboratories #003709) mice were used. A 2 mm-diameter full thickness defect in the spinotrapezius muscle was created as previously described (San Emeterio et al., 2017). Briefly, a longitudinal 1-inch incision as made just after the bony prominence of the shoulder blade. The overlying fascia was dissected away and the spinotrapezius muscle was identified. The edge of the spinotrapezius was reflected and positioned against a sterile piece of wood and a 2 mm biopsy punch was made through the muscle. A 3 mm-diameter nanofiber scaffold implant was placed over the defect and sutured at the top to the muscle with a 10-0 suture. The skin incision was closed with wound clips.

### Tissue Harvest and Flow Cytometry

To collect blood and tissue for flow cytometry analysis, mice were euthanized via CO_2_ asphyxiation. Blood was then collected via cardiac puncture. Red blood cells were lysed in ammonium chloride (1-part blood, 9-parts ammonium chloride) prior to immunostaining for flow cytometry. For analysis of cell composition in spinotrapezius muscles, a 6 mm biopsy punch of muscle tissue centered on the defect was taken and weighed and then digested with 5,500 U/ml collagenase II and 2.5 U/ml Dispase II for 1.5 h in a shaking 37°C water bath. The digested muscles were filtered through a cell strainer to obtain a single cell suspension. Single-cell suspensions were stained for live cells using either Zombie Green or Zombie NIR (BioLegend) dyes in cell-culture grade PBS per manufacturer instructions. Cells were then stained with cell phenotyping antibodies in a 1:1 volume ratio of 3% FBS and Brilliant Stain Buffer (BD Biosciences) according to standard procedures and analyzed on a FACS Aria III flow cytometer (BD Biosciences). The following antibodies were used for cell phenotyping: BV605-conjugated anti-CD4 (BioLegend), BV785-conjugated anti-CD8 (BioLegend), PE-Cy7-conjugated CD3ε (BioLegend), PE-conjugated anti-CD115 (BioLegend), PerCP-Cy5.5-conjugated anti-CD115 (BioLegend), PE-conjugated anti-CD25, FITC-conjugated anti-FoxP3 (eBioscience), BV510-conjugated anti-CD11b (BioLegend), BV421-conjugated anti-CD11b (BioLegend), APC conjugated anti-Ly6C (BioLegend), BV711-conjugated anti-CD64 (BioLegend), PE-conjugated anti-MerTK (BioLegend), APC-Cy7-conjugated anti-Ly6G (BioLegend), PE-Cy7 conjugated anti-CD206 (BioLegend), FITC-conjugated anti-CD206 (BioLegend), PE-Cy5 conjugated anti-CD29 (BioLegend), PerCP-Cy5.5-conjugated anti-CXCR4 (BioLegend), and APC-conjugated Lineage antibody cocktail containing antibodies for mouse CD3e, CD11b, CD45R/B220, Ly-76, Ly6G, and Ly6C (BD Pharmingen). Additionally, 30 μL of AccuCheck Counting Beads (Invitrogen) were added per sample for absolute quantification of cell populations. Single, live cells were selected in FlowJo software for subsequent analysis. Myeloid cells were gated as CD11b^+^ and lymphoid cells as CD3^+^ cells. Unless indicated otherwise, macrophages were gated as CD11b^+^CD64^+^MerTK^+^ cells, where M1-like macrophages were further characterized by their CD206^lo^Ly6C^hi^ signature and M2-like macrophages were characterized with a CD206^hi^Ly6C^lo^ protein signature. Monocytes were gated as CD11b^+^CD64^+^MerTK^–^ cells, with inflammatory monocytes defined as Ly6C^hi^ monocytes and anti-inflammatory as Ly6C^lo^ monocytes. CD3^+^ T cells were gated as CD4^+^ or CD8^+^ T cells based on their high expression of CD4 and CD8, respectively. MuSCs were gated as Lineage negative (Lin^–^) CD29^+^ CXCR4^+^ cells.

### High Dimensional Analysis of Flow Cytometry Data

#### Uniform Manifold Approximation and Projection (UMAP)

Uniform manifold approximation and projection is a non-linear dimensionality reduction algorithm. UMAP is able to embed high-dimensional data into a space of two or three dimensions, and cells are visualized in a scatter plot, where similarity is demonstrated via proximity to other points (Becht et al., 2018). Prior to UMAP dimensional reduction, we pre-gated each flow cytometry sample to select cellular subsets of interest and then imported into Python 3.7 using fcsparser^1^ and Pandas 2.5. Each was normalized by applying arcsinh/150, and UMAP parameters of n_neighbors = 15 and min_dist = 0.1 were applied for compliance with UMAP assumptions. A composite UMAP projection that utilized data points from all desired samples was generated using Matplotlib. Each cell was then phenotyped by overlaying the pre-gated cell subset onto the UMAP projection^2^.

#### Spanning-Tree Progression Analysis of Density-Normalized Events (SPADE)

Spanning tree progression of density normalized events is a visualization tool creating a 2D minimum spanning tree organized into nodes representing clusters of cells similar in their surface marker expressions. The size and color of each node are relative to the number of cells present and the median marker expression (Qiu et al., 2011). SPADE was performed through MATLAB and the source code is available at http://pengqiu.gatech.edu/software/SPADE/. MATLAB-based SPADE automatically generates the tree by performing density-dependent down-sampling, agglomerative clustering, linking clusters with a minimum spanning-tree algorithm and up-sampling based on user input. The SPADE tree was generated by exporting select pre-gated cellular subsets. The following SPADE parameters were used: Apply compensation matrix in FCS header, Arcsinh transformation with cofactor 150, neighborhood size 5, local density approximation factor 1.5, max allowable cells in pooled downsampled data 50000, target density 20000 cells remaining. Number of desired clusters ranged from 60 to 70.

### Whole Mount Immunofluorescence of Spinotrapezius Tissues

Mice were euthanized 14 or 28 days after surgery via CO_2_ asphyxiation. Post-euthanasia, mouse vasculature was perfused with warm saline followed by 4% PFA until tissues were fixed. The entire spinotrapezius muscle was explanted and permeabilized overnight with 0.2% saponin, then blocked overnight in 10% mouse serum. For immunofluorescence, tissues were incubated at 4°C overnight in a solution containing 0.1% saponin, 5% mouse serum, 0.5% bovine serum albumin, and the following conjugated fluorescent antibodies: Alexa Fluor 488 or Alexa Fluor 650 anti-desmin (1:200 dilution, Abcam), Alexa Fluor 594 anti-CD31 (1:100 dilution, BioLegend), Alexa Fluor 650 anti-CD68 (1:200 dilution, Bio-Rad), Alexa Fluor 488 anti-GFP tag (1:200 dilution, BioLegend), rhodamine alpha-bungarotoxin (1:200 dilution, Life Technologies). Following immunostaining, tissues were washed four times for 30 min each in 0.2% saponin for the first two washes, 0.1% saponin for the third wash, and PBS for the final wash, and then mounted in 50/50 glycerol/PBS.

### Imaging and Quantification of Whole-Mount Immunofluorescence

Three-dimensional, tiled scans of whole-mount spinotrapezius muscles were performed on a Zeiss LSM 710 confocal microscope. Immunofluorescence imaging parameters were kept similar across all samples, with adjustments made only to keep image quality similar. Full-thickness Z-stacks (2 μm step size) were collected across the entire x-y plane analyzed. Max intensity projections were generated from 3D confocal images for 2D analysis in Zen software (Zeiss). Second harmonic generated signal was collected via 40× objective using a Chameleon laser to excite tissues at 790 nm and signal collected over 380–420 nm with acquisition parameters kept identical across animals. Desmin, CD68, and SHG signal from 3D z-stacks were rendered in Imaris as surfaces. For desmin surfaces the following parameters were used: auto-smoothing, thresholding set to 14 units, surfaces selected by eye. For SHG surfaces, the following parameters were used: no smoothing, auto-threshold set, all surfaces selected. CD68^+^ surfaces were identified by smoothing with a 1.5 μm grain size and an automatic threshold on absolute intensity. Touching objects were split using a seed points diameter of 13.8 μm. Muscle fiber and collagen alignment (Figure 6) were analyzed using a home-built 2 photon imaging system as described previously (Forouhesh Tehrani et al., 2020; Pendleton et al., 2020). In brief, SHG images (512 × 512 pixels) of collagen and actin muscle structure were produced with linearly polarized excitation rotated at 10° intervals. The SHG signal was normalized and averaged over 20 frames, then color-coded based on the maximum intensity angle of each pixel. Each color serves to represent the dominant orientation of the underlying SHG sensitive structures (e.g., actin/myosin bands and collagen). We captured simultaneous 2 photon fluorescence images of sarcomere structure using Alexa Fluor 488 anti-desmin. The orientation in relation to the direction of the muscle was quantified in four images from each treatment group. Quantification of collagen deposition was performed in ImageJ software. Quantification of 2-dimensional fiber diameter was performed by measuring the widest portion of each regenerated muscle fiber (determined based on the morphology of desmin-positive regions) within the defect region from the day 14 2D maximum intensity projections in Zen software. To quantify fiber alignment, a parallel grid was overlaid on the 2D maximum intensity projection of desmin, following the axis of uninjured fibers. The angle between regenerated fibers (determined based on morphology of desmin positive regions) and the parallel lines in the grid was measured in FIJI. All angles were measured to be between 0 and 90°.

### SPADE Node Heatmap

Spanning tree progression of density normalized events dendrogram heatmaps were constructed with calculated z-scores of fluorescence marker expression intensities for each surface marker used to immunophenotype a specific cell type across all nodes occupied by the cell type. Each row of the heatmap corresponds to a specific surface marker while each column represents an individual annotated SPADE node for a specified cellular subset.

### Statistical Analysis

All statistical analysis was performed in GraphPad Prism software. Data presented as mean ± standard error of the mean (S.E.M.) unless otherwise indicated. For pairwise comparisons, unpaired *t*-test (according to experimental design) with Welch’s correction if variance was significantly different was used. For grouped analyses, one-way ANOVA with Tukey’s post-test was used for multiple comparisons. Unless otherwise noted, *p* < 0.05 was considered statistically significant.

## Data Availability Statement

The original contributions presented in the study are included in the article/Supplementary Material, further inquiries can be directed to the corresponding author/s.

## Ethics Statement

The animal study was reviewed and approved by Georgia Institute of Technology Institutional Animal Care and Use Committee.

## Author Contributions

CS, LH, TS, GW, LM, PQ, YJ, NW, and EB designed the research, analyzed the data, and wrote the manuscript. CS, LH, and TT performed the research and wrote the manuscript. CS, LH, TT, MO, EP, WY, CO, AL, and HL performed research, analyzed the data, and reviewed the manuscript. All authors contributed to the article and approved the submitted version.

## Conflict of Interest

The authors declare that the research was conducted in the absence of any commercial or financial relationships that could be construed as a potential conflict of interest.

## Footnotes

1 https://github.com/eyurtsev/fcsparser

2 https://github.com/lmcinnes/umap
