# Supplementary material for: Nanofiber-Based Delivery of Bioactive Lipids Promotes Pro-regenerative Inflammation and Enhances Muscle Fiber Growth After Volumetric Muscle Loss
Source: Front Bioeng Biotechnol. 2021 Mar 19;9:650289. doi: 10.3389/fbioe.2021.650289 (PMC8017294; doi:10.3389/fbioe.2021.650289)
Supplement: Supplementary file 1 [file Data_Sheet_1.docx]

Supplementary Material

# Supplementary Figures


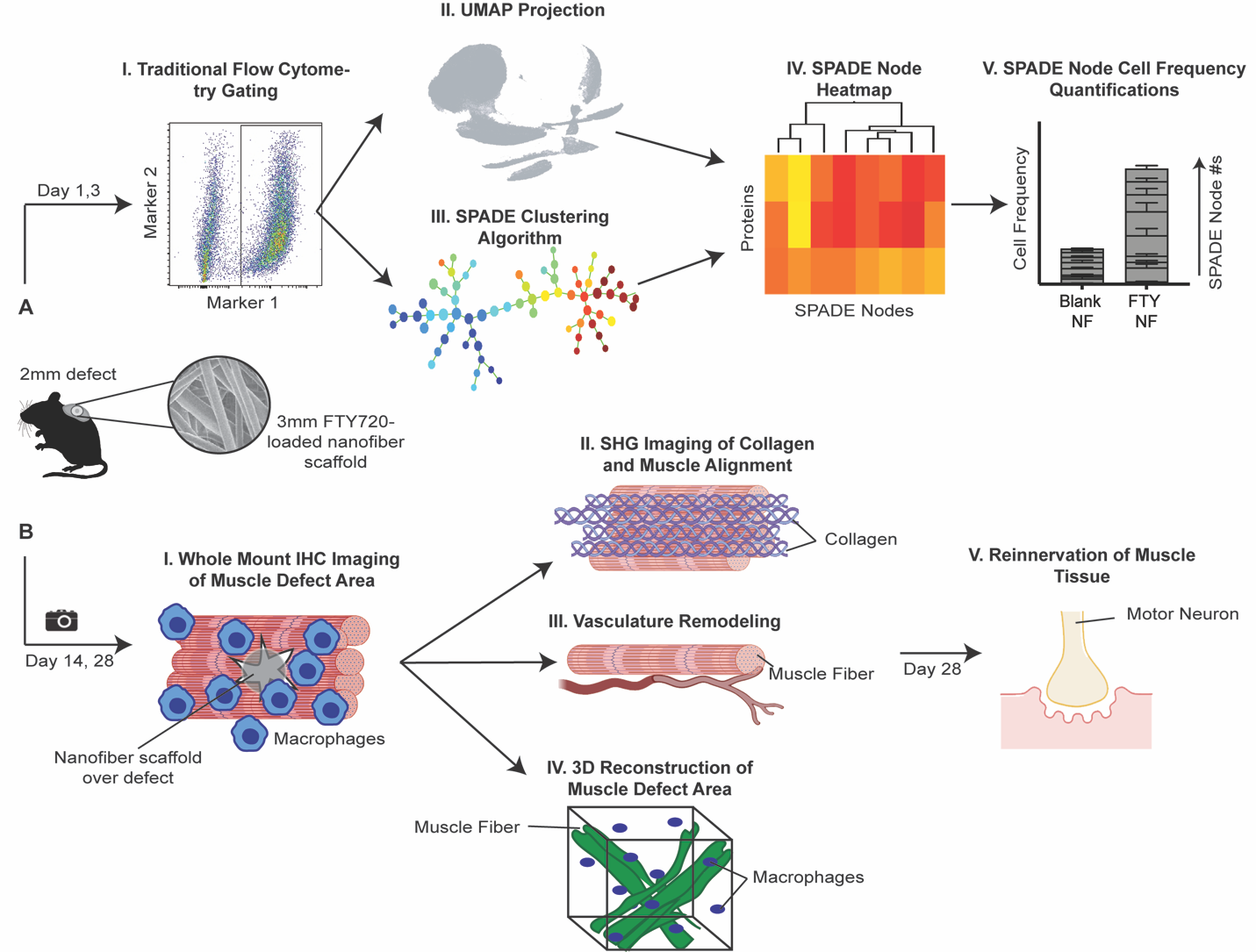


**Fig. S1. Graphical workflow for targeting S1P receptor signaling to modulate local inflammation after VML injury with nanofiber scaffolds.** Graphical representation of the methods utilized within this study, including high dimensional single-cell analysis with flow cytometry **(A)** as well as whole mount immunohistochemistry **(B)** on explanted muscles. A 2mm VML injury was made to the spinotrapezius muscle and then treated with a 3mm nanofiber scaffold (blank or FTY720-loaded). For single cell analysis, our approach includes traditional flow cytometry gating on select subsets (A-I), visualizing cellular heterogeneity with 2D UMAP projections (A-II), utilizing pseudotime trajectory analysis with SPADE (A-III), and representing our data with SPADE generated heatmaps (A-IV) and cell frequency quantifications within annotated SPADE nodes (A-V). Whole mount imaging was utilized to analyze metrics of muscle healing (B-I), where we studied the resulting collagen alignment (B-II), vasculature development (B-III), muscle fiber diameter with 3D surface rendering (B-IV), and reinnervation of the injured muscle tissue (B-V). Created with BioRender.com


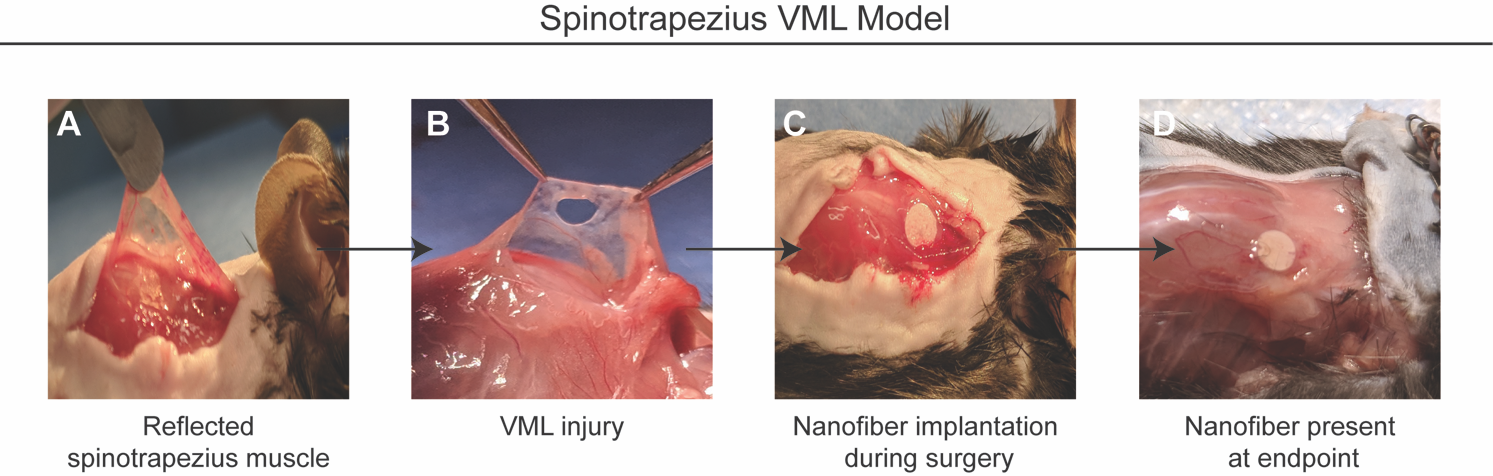


**Fig. S2. Spinotrapezius VML surgical model with nanofiber scaffold implantation.** Surgical procedure steps for VML model. **(A)** Spinotrapezius muscle is isolated from surrounding tissue and reflected. **(B)** A 2mm, full thickness biopsy punch is made through the muscle. **(C)** A 3mm-diameter nanofiber scaffold (blank or FTY720-loaded) was placed over the defect and sutured to the muscle directly following VML injury and then skin is closed with wound clips. **(D)** At pre-determined endpoint following injury, nanofiber scaffold implant remains present and in place over the defect site.


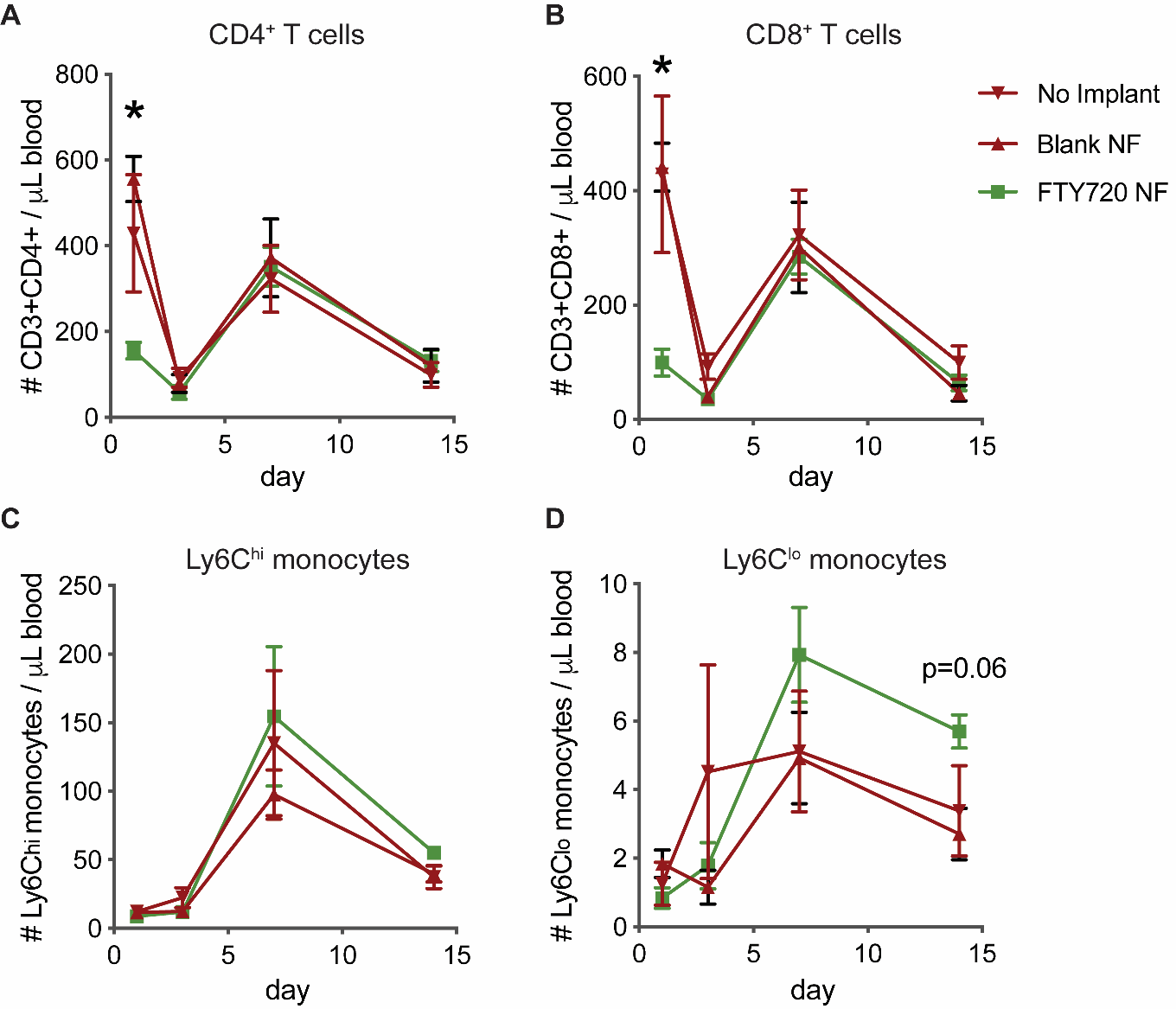


**Fig S3. FTY720-loaded nanofiber implant induces acute lymphopenia following spinotrapezius VML injury.** Immune cell kinetics in the blood at 1, 3, 7, and 14 days post injury. CD4^+^ T cell **(A)**, CD8^+^ T cell **(B)**, Ly6C^hi^ inflammatory monocyte **(C)**, and Ly6C^lo^ anti-inflammatory monocyte **(D)** cell counts at selected timepoints after surgery. Data presented as mean ± S.E.M. Statistical analyses performed by two-way ANOVA with Tukey’s post-hoc test to compare means. *p < 0.05, n= 4-6 animals per treatment group.


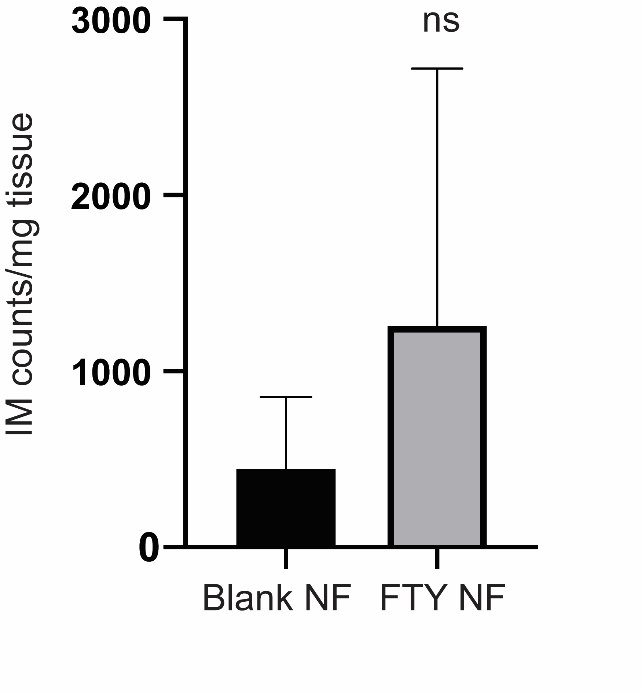


**Fig S4. Infiltration of Ly6C^hi^ inflammatory monocytes into the injured muscle 3 days post injury is not significantly changed with FTY720 treatment.** Flow cytometry analysis of Cd11b^+^CD64^+^MerTK^-^Ly6C^hi^ monocytes in injured spinotrapezius muscle 3 days post injury between mice treated with blank nanofibers and FTY720-loaded nanofibers. Data presented as mean ± SD. Statistical analysis performed by unpaired t-test. n= 8 animals per treatment group.


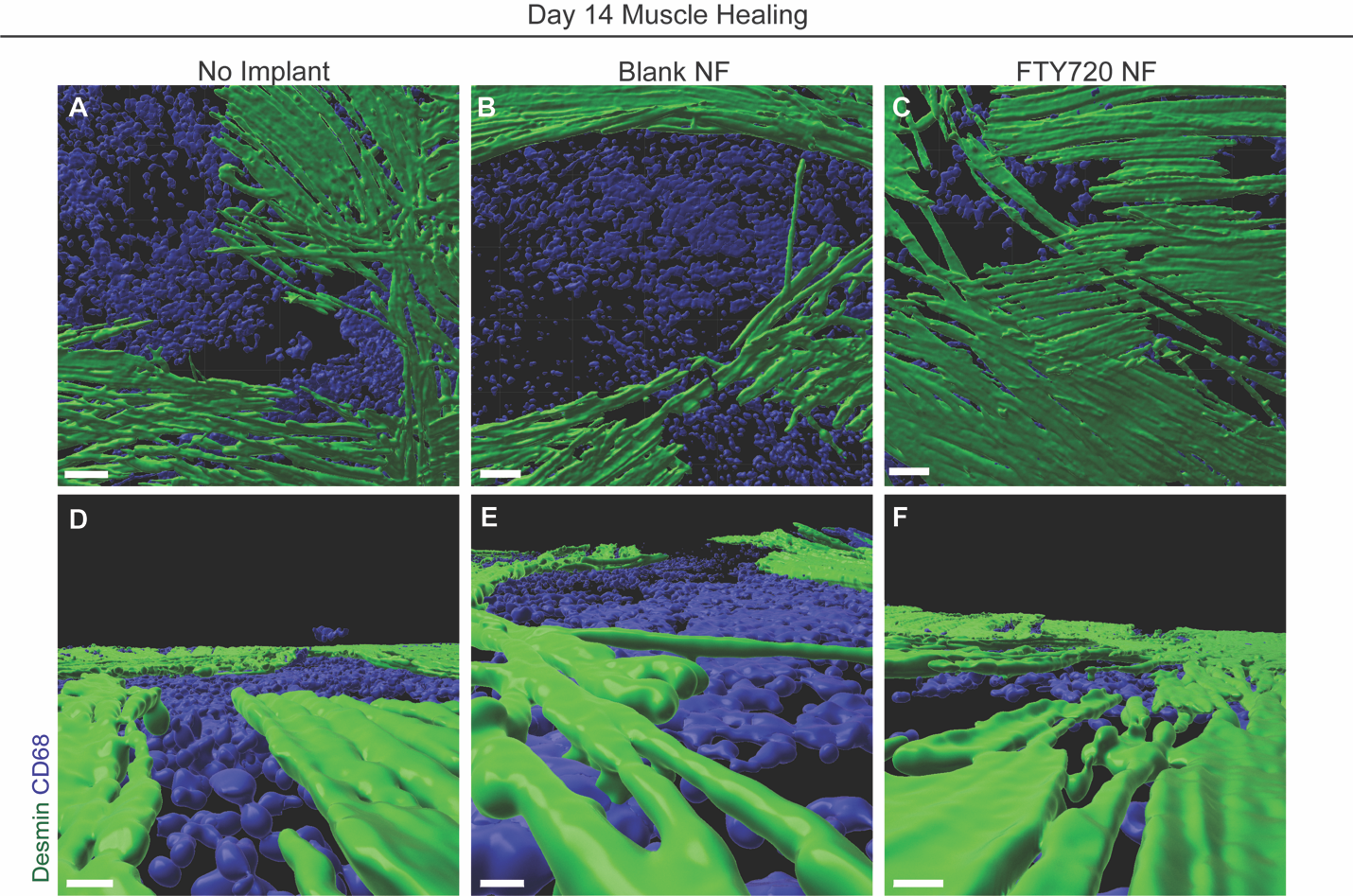


**Fig. S5. Functional antagonism of S1P1 with FTY720 results in improved myofiber bridging across defect.** Representative images of muscle defect area 14 days post injury for each treatment group rendered in Imaris to permit 3D visualization of injury niche. Images stained with desmin (green, muscle fibers) and CD68 (blue, macrophages). Top view of defect area presented in top row to assess myofiber bridging across void area **(A-C)** while side view shown in second row **(D-F)** to visualize CD68^+^ macrophage infiltration into the site of injury. Scale bar 100µm.


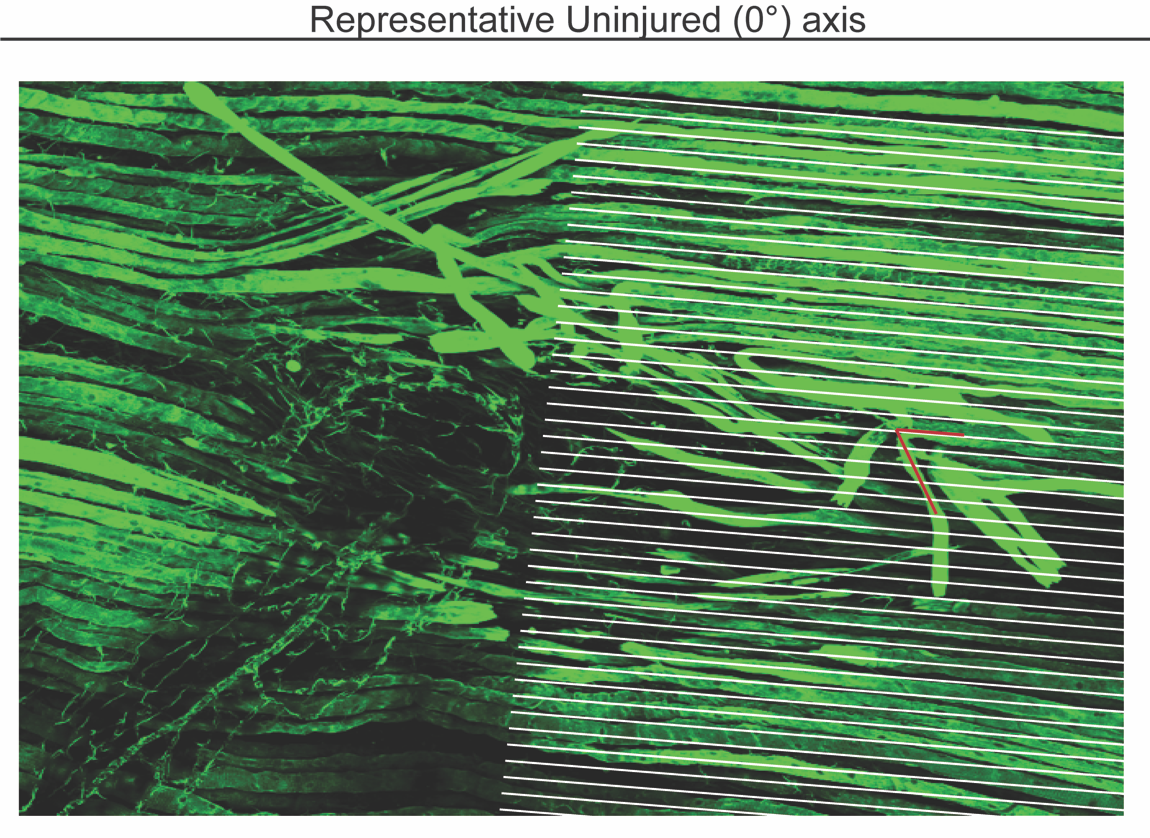


**Fig. S6. Identification of uninjured (0°) muscle fiber axis for fiber alignment analysis.** Representative image of muscle defect 14 days post injury with parallel grid (white lines) overlaid onto image, following the axis of healthy, uninjured fibers. The angle between regenerating muscle fibers and the axis of uninjured muscle fibers was measured (example angle denoted in red). All measured angles range from 0°-90°, where a higher angle measurement indicates poor alignment of the new, regenerating muscle fibers.


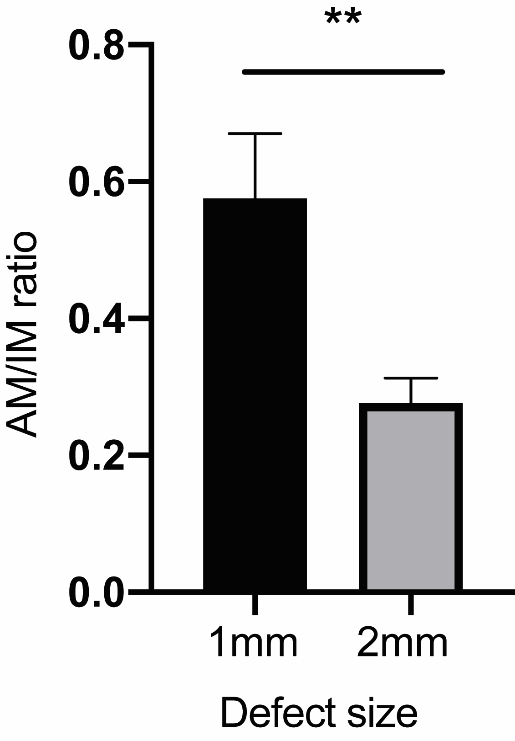


**Fig. S7. Critically sized defect in the spinotrapezius VML model presents dysregulated ratio of pro-regenerative to pro-inflammatory monocytes within injured tissue.** The ratio of Ly6C^lo^ anti-inflammatory monocytes (AM) to Ly6C^hi^ inflammatory monocytes (IM) within the injured spinotrapezius muscle was measured by flow cytometry between subcritical (1mm) defects and critical (2mm) VML defects 3 days after injury with no FTY720 delivery. Critical VML defects (2mm) result in a significantly lower AM/IM ratio compared to injuries of 1mm size. Data presented as mean ± S.E.M. Statistical analysis performed by unpaired t-test. **p<0.01, n= 4 (1mm), 8 (2mm)
